# Repeatability of ovarian antral follicular wave dynamics in women

**DOI:** 10.3389/fendo.2026.1886924

**Published:** 2026-08-17

**Authors:** Gabriella Antaya, Heidi Vanden Brink, Chel Hee Lee, Angela Baerwald

**Affiliations:** 1 Department of Obstetrics and Gynecology, University of Saskatchewan, Saskatoon, SK, Canada; 2 Department of Nutrition, Texas A&M University, College Station, TX, United States; 3 Department of Critical Care Medicine, University of Calgary, Calgary, AB, Canada

**Keywords:** antral follicle count, antral follicular waves, follicular wave, ovarian follicle, repeatability

## Abstract

**Introduction:**

Two or three waves of follicular development have been documented in the human menstrual cycle; however, the repeatability of ovarian follicular wave dynamics across multiple cycles is not known. In the bovine species, the number of follicular waves has been documented to be repeatable in 2/3 of estrous cycles. We tested the hypotheses that the numbers and patterns of ovarian antral follicular waves in women are repeatable across cycles and become less repeatable with age.

**Methods:**

Transvaginal ultrasonography was conducted every 1–3 days over 2 interovulatory intervals (IOIs) for 22 participants. Participants of reproductive age (RA, age 18-35, n=14) and advanced reproductive age (ARA, age 36-55, n=8) were evaluated. The numbers and diameters of follicles ≥2 mm in each ovary were tabulated for each study visit following retrospective review of cineloops. Follicle waves were defined as an increase and subsequent decrease in the number of follicles ≥5 mm, associated with the growth of 2 or more follicles to ≥6 mm. Major waves were defined as a group of follicles from which a dominant follicle was selected for preferential growth. Conversely, in minor waves, selection of a dominant follicle did not occur. The numbers and patterns of follicle waves were compared between cycles and age groups.

**Results:**

The mean number of waves was 2.0 in cycle 1 and 2.1 in cycle, with no differences observed between age groups or cycles (p>0.1). Variability in minor and major wave patterns was reported among women, with no difference between age groups or cycles. Overall, 91% of women exhibited repeatability in the number of waves over the 2 IOIs (20/22 women) while 59% exhibited repeatability in wave patterns (13/22 women). Repeatability in the numbers and patterns of waves did not differ between age groups.

**Discussion:**

Data suggest that the numbers and patterns of antral follicular waves are repeatable across 2 cycles in the majority of women. Research is ongoing to further characterize antral follicular wave dynamics, which may enable personalization of ovarian stimulation therapies.

**Clinical trial registration:**

www.clinicaltrials.gov, identifier NCT01389141.

## Introduction

Ovarian follicles are the structural and functional units of the ovary, which regulate the menstrual cycle. Antral follicles develop in multiple waves throughout the menstrual cycle in women (1–9), similar to the bovine and equine species (10–22). Previous research from our lab has shown that 68% of reproductive age (19–43 years) women exhibited 2 waves of follicle development during an interovulatory interval (IOI) while 32% developed 3 waves (2). In women with 2 waves, the first wave emerged around the time of ovulation followed by emergence of a second wave in the late luteal or early follicular phase (1, 2). In women with 3 follicular waves, on average, the first wave emerged in the early luteal phase, a second wave emerged in the late luteal-early follicular phase, and a third wave developed in the mid follicular phase (1, 2). In all women evaluated, the final wave of the IOI was ovulatory, while all preceding waves were anovulatory (1, 2). A rise in serum FSH appears to precede the emergence of each wave (11, 23). Women with 3 waves have been shown to have a longer cycle (29 days) compared to those with 2 waves (27 days) (2). Follicular waves can be classified as either major or minor (1). A major wave is characterized as a group of follicles from which a dominant follicle is selected for preferential growth (1, 24). Conversely, in minor waves, selection of a dominant follicle does not occur (1).

Follicular wave dynamics have been evaluated in women during the transition to menopause. The development of Luteal Phase Dominant Follicles (LPDFs) has been reported in women of advanced reproductive age (ARA), in association with greater variability in cycle length compared to reproductive age (RA) women (24). Women of advanced reproductive age developed LPDFs which emerged earlier relative to ovulation, grew longer and to larger diameters, sometimes producing atypically high levels of estradiol compared to reproductive age women (24). The presence of LPDFs was also associated with lower concentrations of luteal phase progesterone and inhibin A, which are produced by the corpus luteum (CL) (25, 26). Additionally, a greater incidence of polyovulation as well as periods of no antral follicular development (termed “lag phases”) occurred in advanced versus reproductive aged women (24).

Documentations of ovarian follicular waves have provided a new model for understanding the physiology of the human menstrual cycle. However, there is still much that remains unknown. Research to characterize follicular wave dynamics in women to date has been limited to a single cycle, with no knowledge of whether the numbers and patterns of follicular waves are consistent across multiple cycles. Initial studies to examine the repeatability of follicular waves in the bovine species were conflicting (12, 27, 28). However, a more recent study by Jaiswal et al., revealed that the number of follicular waves across bovine estrous cycles was highly (70%) repeatable (14). Knowledge of the repeatability of ovarian follicular wave dynamics in domestic farm animal species has been applied to the development of protocols for ovarian synchronization, superstimulation, and fixed-time artificial insemination (15).

The understanding of ovarian follicular waves in women has been applied to the development of novel ovarian stimulation regimens, including luteal phase stimulation (29), random start stimulation (30), and double stimulation (31, 32). Synchronizing ovarian stimulation with wave emergence in poor responder patients undergoing conventional follicular phase ovarian stimulation has been shown in a single study to increase the number of dominant follicles that develop and serum estradiol concentrations, but not fertilization or live birth rates (33); further confirmatory studies are required. The effects of synchronizing stimulation with wave emergence in novel luteal phase and double stimulation protocols, however, are not known. Through personalizing ovarian stimulation to each woman’s unique cycle characteristics (eg. day of wave emergence), we may be able to improve clinical outcomes in poor responder patients.

The overall objective of this study was to characterize the repeatability of ovarian follicular wave dynamics across 2 IOIs in women. The first objective was to compare the number of follicular waves across 2 IOIs, while the second objective was to characterize major and minor follicular growth patterns across the 2 cycles. Thirdly, we aimed to determine whether repeatability in follicular wave dynamics across 2 IOIs differed with age. We tested the hypotheses that the numbers and patterns of ovarian antral follicular waves in women are repeatable across cycles and become less repeatable with age.

## Materials and methods

A prospective, observational study was conducted. The study protocol was approved by the Biomedical Research Ethics Board at the University of Saskatchewan and the Strategic Priorities and Planning Committee in the Saskatchewan Health Authority. Study procedures complied with the guidelines of the Tri-Council Policy Statements on the Ethical Conduct for Research Involving Humans. Informed consent was obtained from all participants before study procedures were initiated.

Study participants were recruited via paper and/or electronic posters advertised within the Saskatchewan Health Authority and University of Saskatchewan. Inclusion criteria included: individuals of born biologic female sex; 18–55 years of age; and normal complete blood count, serum TSH, prolactin and β-hCG. In women 18–35 years of age, additional criteria included a history of regular menstrual cycles (i.e., 21–45 days in length). In contrast, women 36–55 years of age were required to have a history of less than 12 months of amenorrhea. Exclusion criteria for all participants comprised: hormone therapy of any kind within 3 months of the study start date, medical conditions or medications known to interfere with reproductive function, participation in an interventional drug trial within 30 days of the study, current smoking, pregnancy, lactation, presence of only one ovary, and ovaries unable to be visualized ultrasonographically.

Ultrasound images from 22 participants were available for evaluation in this study. Data regarding follicular wave dynamics and hormone production in 1 of the 2 cycles were reported previously (24). Participants 18–35 years of age were classified as reproductive age (RA; n=14), and those 36–55 years of age were defined as advanced reproductive age (ARA; n=8). Within both RA and ARA groups, only ovulatory menstrual cycles were included in analyses Women with lag phases (i.e., no follicular activity for >20 days) were excluded from analyses.

Transvaginal ultrasonography was conducted every 1–3 days across 2 IOIs using a 2-Dimensional (2D) B-mode imaging system (6-9MHz curvilinear array transducer, Ultrasonix RP, Burnaby, BC, Canada) (24). At each study visit, 2D cineloops of each ovary were captured in both sagittal and transverse planes. The numbers and diameters of follicles ≥2 mm in each ovary were tabulated for each study visit following retrospective review of cineloops (Santesoft DICOM Editor Version 3.1.0, MicroDicom Viewer Version 3.4.7; IC Measure Version 2.0.0.245). Follicle diameter was measured at the largest cross-sectional area of each follicle in the cineloop. The mean maximal diameter was calculated as the average of follicle length versus width, with measurements made perpendicular to each other. A consistent plane of imaging (i.e., sagittal or transverse) was used within each participant for retrospective analysis of follicle dynamics throughout the IOI.

Follicle growth profiles over the 2 IOIs were created using the Identity (ID) Method (1, 19, 20). The location, size and shape of each follicle was sketched each day throughout the 2 IOIs. The growth profiles of individually identified follicles >6 mm from the day of ovulation backwards to the day of emergence were then created. Day of emergence (DOE) was defined as the day in which the largest follicle of the wave was first detected a diameter of 4–5 mm. Growth profiles of individually identified follicles >6 mm over the 2 IOIs were graphed (Microsoft Excel Version 2023). The numbers of follicles within each ovary at each study visit were tabulated and graphed for the following diameter categories: 2–5 mm, 2–10 mm, ≥4 mm, ≥5 mm and 4–6 mm. The total number of follicles for both ovaries was tabulated (antral follicle count, AFC).

Follicle waves were defined as an increase and subsequent decrease in AFC ≥5 mm, associated with the growth of 2 or more follicles to ≥6 mm. In 3/8 participants in the ARA group, a wave was characterized by a rise and fall in AFC in association with only 1 follicle to >6 mm. These 3 participants exhibited a lower number of follicles per ovary and follicles developing to < 6 mm appeared to emerge within the wave. A major follicle wave was defined as a wave in which a dominant follicle (DF) developed. A DF was defined as a follicle reaching a diameter of ≥10 mm and exceeding the next largest follicle by ≥2 mm. A minor follicular wave was defined as one in which no dominant follicle developed.

Normality and equality of variance were evaluated for each study endpoint using Shapiro-Wilks and Levene’s tests, respectively (SPSS Version 28.0, 2021). Wilcoxon’s Signed Rank tests were used to compare differences between proportions of numbers and patterns of waves between the two cycles (SPSS Version 28.0, 2021). Fishers’ Exact tests were conducted to compare repeatability among numbers and patterns of follicle waves between age groups (SPSS Version 28.0, 2021). The Mann-Whitney U test was performed to compare the mean number of waves and mean day of emergence (DOE) for each wave between the 2 cycles (SPSS Version 28.0, 2021). Mean DOE between age groups was compared using the Kruskal Wallis test (SPSS Version 28.0, 2021). All statistics were conducted with a significance level of 0.10, which was deemed acceptable for the relatively small sample size.

## Results

### Participant demographics

Participant demographics and cycle characteristics between age groups are presented in **Table 1**. The mean age (range) was 27.0 (20.0-35.0) in the RA group and 43.5 (36.0-50.0) in the ARA group. Mean cycle length (range) in the RA group (28.0 days; 21.0-38.0) days was greater than that in the ARA group (24.5 days, 21.0-35.0 days). Gravidity and parity increased with age (p<0.10), as expected. The IOI and follicular phase lengths were shorter in the advanced reproductive vs reproductive age group (p<0.10). Luteal phase length, smoking status and BMI were not different between age groups (p>0.10).

**Table 1:** Participant demographics and cycle characteristics compared between age groups.

	Reproductive age n=14	Advanced reproductive age n=8	P-value
Age (years)	27.0 (20.0-35.0)	43.5 (36.0-50.0)	0.00
BMI (kg/m^2^)	25.3 (21.1-31.0)	24.9 (21.7-30.9)	0.87
Gravidity (#)	0.0 (0.0-3.0)	3.0 (2.0-5.0)	0.00
Parity (#)	0.0 (0.0-3.0)	2.5 (2.0-4.0)	0.00
Ever-smoker (n)	1/14 (7%)	3/8 (38%)	0.53
Cycles combined			
IOI Length (days)	28.0 (21.0-38.0)	24.5 (21.0-35.0)	0.02
Follicular Phase Length (days)	14.0 (11.0-28.0)	11.0 (9.0-20.0)	0.00
Luteal Phase Length (days)	14.0 (5.0-17.0)	14.0 (11.0-16.0)	0.54
Cycle 1			
IOI Length (days)	27.0 (21.0-31.0)	24.5 (22.0-33.0)	0.05
Follicular Phase Length (days)	13.0 (11.0-17.0)	11.0 (9.0-19.0)	0.02
Luteal Phase Length (days)	14.0 (5.0-16.0)	13.5 (11.0-15.0)	0.44
Cycle 2			
IOI Length (days)	29.0 (22.0-38.0)	24.5 (21.0-35.0)	0.15
Follicular Phase Length (days)	14.0 (11.0-28.0)	11.5 (10.0-20.0)	0.04
Luteal Phase Length (days)	14.0 (7.0-17.0)	14.0 (11.0-16.0)	1.00

Day 0= day of ovulation #1 of the IOI.

Data are shown as median (range).

In 77% of participants (17/22), the 2 IOIs were consecutive; in the remaining 23% of participants (5/22), cycles were not consecutive. There were no differences in the repeatability of numbers or patterns of follicular waves in women with continuous versus discontinuous cycles (P>0.10); therefore, data were combined.

### Repeatability in the numbers of follicular waves

**Figure 1:**
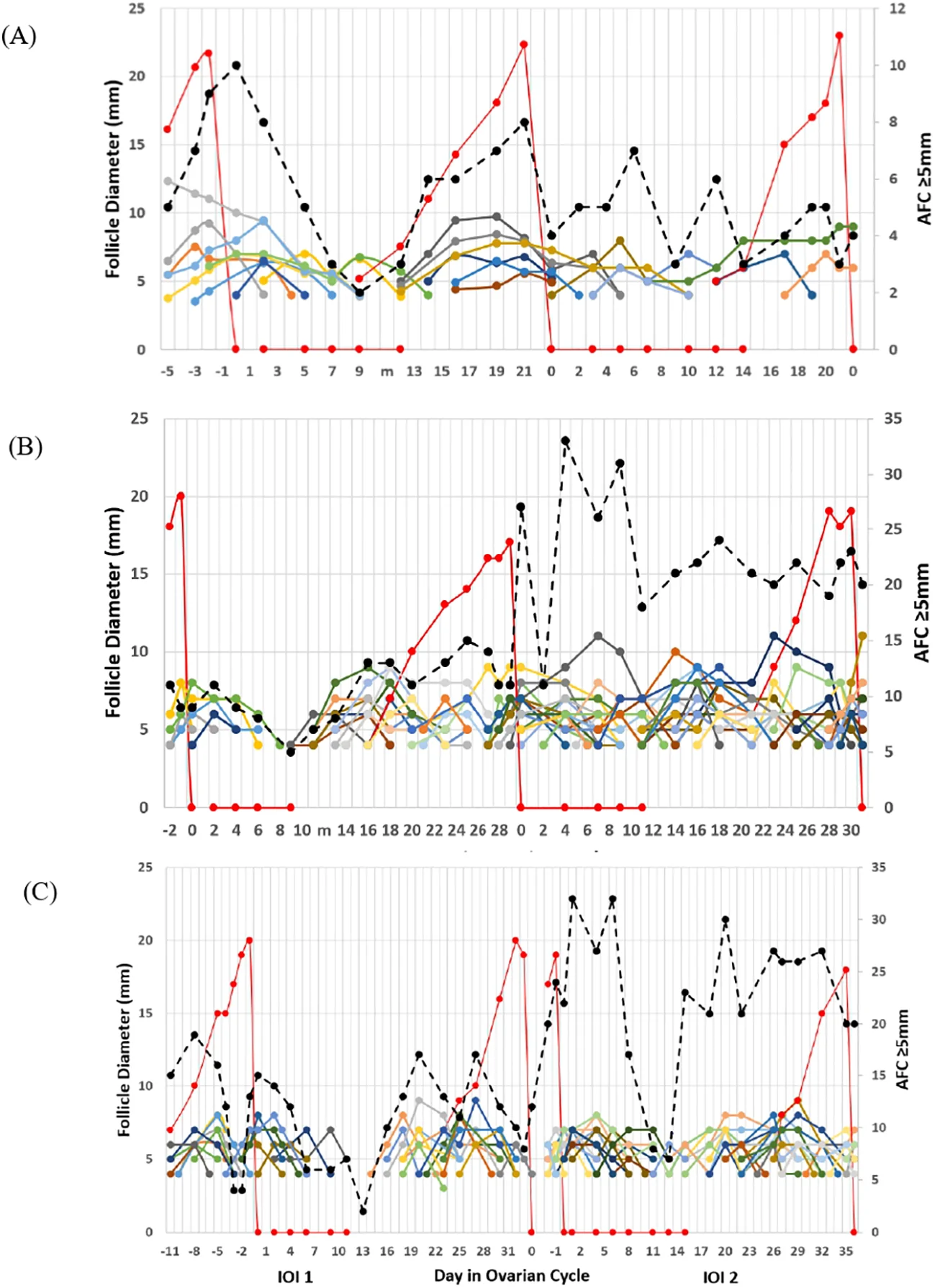
**(A–C)** An example of a follicular growth profile for an advanced reproductive age woman with a 2 wave (minor-major) pattern in the first IOI and a 2 wave (minor-major) pattern in the second IOI **(A)**. An example of a follicular growth profile for an advanced reproductive age woman with a 2 wave (minor-major) pattern in the first IOI and a 3 wave (major-minor-major) pattern in the second IOI **(B)**. An example of a follicular growth profile for a reproductive age woman with a 2 wave (minor-major) pattern in the first IOI and a 3 wave (minor-minor-major) pattern in the second IOI **(C)**. Data were centralized to the day of ovulation #1. The red lines represent ovulatory follicles. The solid red line along the x-axis represents the days when the CL was visualized. The black, dotted line depicts AFC ≥5mm. All other colored lines represent individual follicles within each wave (determined using the ID Method).

Examples of follicular growth profiles are shown in **Figure 1**. In **Figure 1A**, follicular growth profiles for a reproductive age woman with a 2 wave (minor-major) pattern of follicular development in both cycles 1 and 2 are shown. By comparison, in **Figure 1B**, follicular growth profiles for a reproductive age woman with a 2 wave (minor-major) pattern in the first cycle and 3 wave (major-minor-major) pattern in the second cycle are shown. In **Figure 1C**, follicular growth profiles for an advanced reproductive age woman with a 2 wave (minor-major) pattern in the first cycle and 3 wave (minor-minor-major) pattern in the second cycle are shown. Comparisons of the mean number of follicular waves in cycles 1 and 2 are shown in **Table 2**. Among all women, the mean number of follicle waves was 2.1. There were no differences in the mean number of waves between age groups (p>0.10, **Table 1**). As there were no differences in mean number of waves between cycle 1 and 2 (p=0.16), data were combined for subsequent analyses (cycles, n=44).

**Table 2:** Numbers and patterns of follicular waves.

Mean number of waves*	All participants (n=22)	Reproductive age (RA, n=14)	Advanced reproductive age (ARA, n=8)	P-value
Cycle 1	2.0 ± 0.0	2.0 ± 0.0	2.0 ± 0.0	1.00
Cycle 2	2.1 ± 0.1	2.1 ± 0.1	2.1 ± 0.1	0.87
P-value	0.16	0.32	0.32	
Proportions of number of waves				
2	42/44 (95%)	27/28 (96%)	15/16 (94%)	0.60
3	2/44 (5%)	1/28 (4%)	1/16 (6%)	0.60
**Repeatability of Wave Numbers**	20/22 (91%)	13/14 (93%)	7/8 (88%)	0.61
Proportions of wave patterns				
Minor-major	33/44 (75%)	21/28 (75%)	12/16 (75%)	0.64
Major-major	9/44 (20%)	6/28 (21%)	3/16 (19%)	0.58
Major-minor-major	1/44 (2.5%)	1/28 (4%)	0/16 (0%)	0.64
Minor-minor-major	1/44 (2.5%)	0/28 (0%)	1/16 (6%)	0.64
**Repeatability of Wave Patterns**	13/22 (59%)	9/14 (64%)	4/8 (50%)	0.42

*Data are shown as mean ± SE.

The numbers, patterns, proportions and repeatability of follicular waves are shown in **Table 2**. The mean number of follicle waves was 2.0 in cycle 1 and 2.1 in cycle 2; the mean number of waves did not differ across the 2 cycles or age groups (p>0.10). Overall, 42/44 (95%) cycles were characterized as having 2 follicular waves and 2/44 (5%) cycles were characterized as having 3 waves; no differences in the proportions of 2 and 3 wave cycles were observed between age groups (p=0.60). Overall, 20/22 (91%) women exhibited repeatability in the numbers of follicular waves (i.e., 2 or 3 waves) between cycles. We found that 13/14 (93%) participants in the reproductive age group and 7/8 (88%) participants in the advanced reproductive age group exhibited repeatability, with no difference between age groups (P = 0.61).

The mean days of wave emergence (DOE) for women with 2 and 3 waves are shown in **Table 3**. For reproductive age women with 2 waves, on average, wave 1 emerged on day 2 and wave 2 emerged on day 14, relative to ovulation #1. By comparison, in advanced reproductive age women with 2 waves, wave 1 emerged on day -2, and wave 2 emerged on day 13, on average. No differences in mean days of wave emergence were detected between age groups (P>0.10). For the one reproductive age woman with 3 waves, wave emergence occurred on days 1, 7 and 18. For the one advanced reproductive age woman with 3 waves, wave emergence occurred on days -1, 15 and 26.

**Table 3:** Mean IOI length and days of wave emergence in women with 2 versus 3 follicular waves.

	Mean IOI length (days)	Day of wave emergence			
		All participants (n=22)	Reproductive age (RA, n=14)	Advanced reproductive age (ARA, n=8)	P-value
2 waves (n=42)	27 ± 0.6				
Wave 1		-2 ± 0.3	-2 ± 0.4	-2 ± 0.7	0.93
Wave 2		14 ± 0.5	14 ± 0.7	13 ± 0.6	0.77
3 waves (n=2)	30, 35				
Wave 1			-1	-1	–
Wave 2			7	15	–
Wave 3			18	26	–

Data are shown as mean ± SE.

Day 0=day of ovulation #1 of the IOI.

Individual data, rather than mean data ± SE, are shown for women with 3 waves where n=2.

### Repeatability in the patterns of follicular waves

The proportions and repeatability of participants exhibiting different major-minor patterns of follicular waves are shown in **Table 2**. Overall, 21/28 (75%) cycles in reproductive age women and 12/16 (75%) cycles in advanced reproductive age women contained a minor-major pattern of follicle growth. In 6/28 (21%) cycles in the reproductive age group and 3/16 (19%) cycles in the advanced reproductive age group, a major-major pattern of follicle growth was detected. In 1/28 (4%) cycles in the reproductive age group and 0/16 (0%) cycles in the advanced reproductive age group, a major-minor-major pattern of development occurred. Finally, 0/28 (0%) cycles in reproductive age women and 1/16 (6%) of cycles in advanced reproductive age women had a minor-minor-major pattern. The patterns of follicle wave dynamics were not different between age groups (P>0.10). Irrespective of age, patterns of follicular wave dynamics were repeatable in 13/22 (59%) women between the 2 cycles. Repeatability in follicular wave patterns occurred in 9/14 (64%) reproductive age women and 4/8 (50%) advanced reproductive age women; proportions were not different between age groups (P>0.10).

## Discussion

The findings from this research suggest that the numbers and patterns of follicular waves are repeatable over 2 cycles in the majority of women, irrespective of age, which supported our primary hypothesis. Overall, the numbers of follicular waves within participants were highly repeatable (91%) across 2 cycles. Our findings are similar to those previously reported in the bovine species in which 70% of cycles exhibited repeatability in numbers of follicle waves during the estrous cycle (14).

In the majority of participants (95%), 2 waves developed within each cycle, while a minority developed 3 waves (5%). The 5% prevalence of 3 wave cycles in the current study was lower than that previously published (32%) (1, 2). It is plausible that a sample size of only 22 women was insufficient to adequately detect 3 wave cycles, as previously reported (1, 2). Also, in an attempt to reduce burden to participants, we were advised to consider a scanning frequency of less than once daily. The current study design of conducting ultrasound exams every 1–3 days was found to make it more difficult to characterize waves, and may have further contributed to the difference in proportions of 2 versus 3 wave cycles in the present versus previous studies from our group.

When data were stratified by age, no differences in repeatability between groups were detected (94% for RA and 88% for ARA), which did not support our hypothesis. We predicted that repeatability would decrease as women age, because it is well established that AFC decreases as women age and as a result, cycle length, follicle growth dynamics, and hormone production becomes more variable (34). The influence of age on repeatability of follicular wave dynamics in domestic farm animals has not been reported, to our knowledge. Given the sample size of only n=8 in the advanced reproductive age group, continued research is required to confirm our findings before results are generalizable.

The greatest proportion of cycles were characterized as having a 2 wave, minor-major pattern (75%), which was consistent with previous studies in our lab (1). The types of 3 wave patterns also differed from those previously reported (1). In the present study, minor-minor-major and major-minor-major 3 wave patterns, but not minor-major-major and major-major-major patterns, were detected (1). Inconsistencies in follicle wave patterns between studies were partly attributed to a small sample size as well as a sampling frequency of every 1–3 days. It is likely that a larger sample size and daily scanning frequency would have increased the likelihood of detecting greater variability in patterns of follicular waves, specifically greater detection of minor waves (which are more prevalent in 3 vs 2 wave cycles). Documentations of major and minor follicular wave patterns in women in the present study are similar to those documented in mares (35).

In this study, we demonstrated that major and minor follicular wave patterns were repeatable in more than half of participants (59%). Our conclusions were based on evaluating follicles which grew to ≥ 6 mm in diameter and the numbers of follicles >5 mm, to maintain consistency with previous documentations of ovarian follicular waves Interestingly, when we attempted to characterize changes in the number of follicles 2–10 mm across cycles, we found preliminary evidence of additional waves. In 14/44 (32%) cycles, we detected a wave (either major or minor) underlying the development of the dominant ovulatory follicle in the mid-late follicular phase. These ‘underlying’ waves often emerged at the time of selection of the dominant follicle in the follicular phase and began to regress prior to the emergence of the next wave. Follicle waves underlying the growth of the preovulatory dominant follicle have been previously described in equine species (35). The physiologic significance of waves underlying the growth of major ovulatory waves in women is not understood. Research is ongoing in our laboratory to further explore changes in the number of follicles 2–5 mm and 2–10 mm across the cycle using daily data as opposed to data every 1–3 days in the present study.

The timing of emergence of each wave during 2-wave cycles was consistent with previous literature (2). When age groups were combined, the first wave of the IOI emerged 2 days prior to ovulation, on average (range=-9 to +2), and the second wave emerged at the time of the luteal-follicular transition (day 14, range= day 8 to 24). We attributed this wide range in day of wave emergence to increasing variability in cycle length as women age, especially in the follicular phase (36). During earlier stages of the menopausal transition, the follicular phase becomes shorter due to high FSH levels which have been shown to accelerate follicular phase recruitment and development (36). Our data supported the notion of a shorter follicular phase as women age (RA = 13.0-14.0 days, ARA = 11.0-11.5 days) (P<0.10). In comparison, the luteal phase length is less variable with age due to the consistent growth of the CL for 6–7 days following ovulation, followed by regression in the absence of conception (37). In the present study, we were only able to detect 3-wave cycles in 2 women. Thus, further research is required in a larger sample of women of various ages to characterize consistency in the timing of wave emergence across multiple cycles. Collectively, the findings from the present study and previous studies (1, 2, 24) support the notion that there is a great degree of variability in both the timing and growth characteristics of follicular waves among women.

The biggest limitation of our study was the small samples size. Although we evaluated only 22 participants, we were able to obtain ultrasonographic data every 1–3 days over 2 complete IOIs in all participants. With this approach, we obtained a large imaging dataset in a relatively small sample. Studies to characterize serial follicular growth over multiple cycles in women are very time consuming and expensive to conduct. Although it would have been preferrable to evaluate a larger sample over >2 cycles, it is very difficult to recruit participants for such studies. From an analytical perspective, it is also extraordinarily time consuming to track the growth of individually-identified antral follicles across multiple menstrual cycles. In addition, humans are known to have a greater number of antral follicles 2-5mm compared to domestic farm species (35), which complicates the detection of follicular waves in women. This is the only study, to our knowledge, to characterize repeatability of follicular waves in women. Given that we were able to detect repeatability in a relatively small sample of women, it is likely that our findings of repeatability would be replicated in a larger sample. An additional limitation of our study was that 2 different investigators were required to retrospectively review images and collect data due to the extended period of time required for data collection and analyses. While standardized approaches for viewing cineloops and measuring/counting follicles were employed between the 2 investigators, it is likely that a certain degree of inter- and intra-observer variability existed. It is possible that advancements in AI-mediated automated detection versus manual mapping of ovarian follicles over time will allow for more practical and efficient data collection in a larger sample of participants (38). Lastly, BMI was controlled for in the present study, as confirmed by similar BMI in the 2 age groups. It is plausible that BMI may impact follicular wave dynamics. Further research in women with elevated versus normal BMI, and/or Polyendocrine Metabolic Ovarian Syndrome (PMOS), is warranted (39).

Evidence of follicular waves has had clinical applications for optimizing assisted reproductive therapies (40–44). Novel ovarian stimulation strategies have been widely adopted, including luteal phase stimulation, double stimulation and random start stimulation (29–33). Understanding whether follicular wave dynamics are repeatable in women is important for providing personalized approaches to ovarian stimulation. Our results support the notion that the timing of ovarian stimulation should not be altered in a given woman, based on age alone. Furthermore, preliminary data suggest that synchronizing ovarian stimulation with wave emergence in poor responder patients undergoing conventional follicular phase ovarian stimulation improves the number of dominant follicles that develop and serum estradiol concentrations, but not fertilization or live birth rates (33). However, the benefits of synchronizing ovarian stimulation with wave emergence during the use of follicular phase, luteal phase, or double stimulation protocols requires continued investigations.

## Conclusion

In summary, the numbers and patterns of antral ovarian follicular wave dynamics are highly repeatable across 2 cycles in women, irrespective of age. Further research is required to elucidate the potential benefits of synchronizing ovarian stimulation with wave emergence for optimization of clinical assisted reproductive outcomes.

## Data Availability

The original contributions presented in the study are included in the article; further inquiries can be directed to the corresponding author/s.
